# Patients’ care dependency in mental health care: Development of a self‐report questionnaire and preliminary correlates

**DOI:** 10.1002/jclp.22574

**Published:** 2018-01-10

**Authors:** Naline Geurtzen, Ger P. J. Keijsers, Johan C. Karremans, Giel J. M. Hutschemaekers

**Affiliations:** ^1^ Radboud University Behavioural Science Institute; ^2^ NijCa^2^re; ^3^ Department of Clinical Psychological Science University Maastricht

**Keywords:** care dependency, psychological treatments, side‐effects, symptoms, therapeutic alliance

## Abstract

**Objectives:**

Patients’ dependency on the therapist or treatment has received little empirical attention. To examine care dependency, we aimed to develop a theory‐driven questionnaire based on three hypothetical dimensions (passive–submissive dependency; active‐emotional dependency; and lack of perceived alternatives) and to provide a preliminary exploration of several correlates of care dependency.

**Method:**

Care dependency, perceived social support, therapeutic alliance, remoralization, and symptom severity were measured in a large cross‐sectional sample of 742 outpatients with various psychiatric disorders. Test–retest reliability was established in a smaller patient sample.

**Results:**

Findings indicated a reliable questionnaire measuring three unidimensional subscales of care dependency (i.e., submissive dependency, need for contact, and lack of perceived alternatives; α’s .74, .81, and .86 respectively; *r*_t1,t2_’s .78, .76, and .80, respectively). These subscales were all positively correlated with each other and with patients’ self‐proclaimed care dependency, but divergent from patients’ trait dependency and symptoms of a dependent personality disorder. Moreover, higher levels of care dependency were correlated with lower levels of remoralization and more symptoms severity, and with a better therapeutic alliance.

**Conclusions:**

A reliable and valid questionnaire was developed to measure patients’ care dependency. Future studies are needed to determine whether care dependency covers an unwanted side‐effect or a crucial ingredient of an effective treatment.

It is generally acknowledged that patients who receive psychological treatment in mental health care can be dependent on their therapist or treatment (see Bornstein, 2005). Less accepted, however, is the idea that professional mental health care treatments actually can increase dependency and make patients dependent. Several recent empirical studies stress the relevance of this idea. For example, Leitner et al. (2013) identified risky developments during the psychotherapeutic process. One of these developments was described as “dependency/isolation,” the degree to which patients feel the therapist to be the most important person in their life or the only one to trust. A total of 18% of the adult patients or ex‐patients participating in their study reported to feel or to have felt highly dependent on their therapist. Also Parker, Fletcher, Berk, and Paterson (2013) showed that one adverse effect of psychotherapy was “idealization of the therapist,” referring to the patient feeling dependent on the therapist, or handling over control to the therapist.

The notion that patients’ care dependency can be induced and reinforced by mental health care treatment is not new. For example, sociohistorical literature considers dependency as a possible side‐effect of a long‐term stay in a psychiatric hospital, sometimes referred to as the “hospitalization‐syndrome” (e.g., Goffman, 1961). More recently, it has been suggested that the mental health care context can elicit patients’ dependency on professional treatments. For example: as a result of the identification of new psychological disorders by mental health care professionals, or the institutions that make help available on demand for 24/7, or the need of psychotherapists to be needed (e.g., Berk & Parker, 2009; Bressi Nath, Alexander, & Solomon, 2012; Bystedt, Rozental, Andersson, Boettcher, & Carlbring, 2014; Clemens, 2010; Hutschemaekers, 2001; Leitner et al., 2013; Linden, 2013; Tait, 1997).

Yet, the idea that the mental health care context can contribute to patients’ care dependency is not widely accepted or at least has received very little theoretical and empirical attention. Instead, patients’ dependency on mental health care often is attributed to personality characteristics of the patient (e.g., Bornstein, 2005, 2011, 2012; Hirschfeld et al., 1977; Morgan & Clark, 2010) or to personality disorders (American Psychiatric Association, 2013). From this perspective, patient dependency has a negative connotation, in that it is often considered as a burden for therapists, and that it could undermine treatment and possibly causes treatments to continue longer than “necessary” (e.g., Bornstein, 2005). However, it is not without debate whether dependency is a negative aspect of treatments (Clemens, 2010; Gardner & Helmes, 2007; Leitner et al., 2013; Parker et al., 2013; Tsai, Desai, & Rosenheck, 2012), or whether dependency is actually helpful or necessary for psychological treatments to become effective (Clemens, 2010; Linden, 2013; Tait, 1997). Various theoretical perspectives, for example attachment theory (Bowlby, 1988), suggest that dependency or health seeking behaviors are likely to arise in short‐term and long‐term therapeutic relationships and could be seen as functional in times of distress (Tait, 1997). Berk and Parker (2009) called this the Scylla–Charybdis dilemma and argued that “some degree of dependence is necessary in the psychotherapeutic relationship to allow the healing common factors to produce their benefits–but its potential to undermine self‐mastery is substantive, can occur early and risks increasing over therapy” (p. 790).

Care dependency thus seems a potentially important factor in psychological treatments. However, to examine whether care dependency is indeed distinguishable from the trait approach to dependency and to find out whether patients’ dependency in mental health care should be seen as a negative side‐effect or as a functional aspect of the therapeutic alliance, an instrument is needed which measures the construct accurately. To the best of our knowledge, there are no existing questionnaires that measure patients’ care dependency in mental health care directly. Hence, the first goal of the current study was to develop a theory‐driven questionnaire of care dependency. To do so, we formulated a provisional definition of care dependency by describing three theoretical dimensions of care dependency, based on which we developed a self‐report questionnaire. The second goal of the current study was to provide a preliminary exploration of some correlates of care dependency that are relevant for clinical practice.

## 1 THREE HYPOTHETICAL DIMENSIONS OF CARE DEPENDENCY

Based on the extant relevant literature, we propose three dimensions of care dependency: passive–submissive dependency, active‐emotional dependency, and the lack of perceived alternatives. Traditionally, dependency has been described in terms of passivity and submissiveness of patients (see Bornstein, 2005). Dependent patients are seen as docile, timid, dull, apathetic, and weak. In addition, dependent patients fail to take much initiative in their treatment and show a submissive and helpless stance (e.g., Birtchnell, 1984; Blatt & Homann, 1992; Goffman, 1961; Tait, 1997). In line with the two‐factor model resulting from Morgan and Clark's (2010) exploratory factor analysis on multiple trait‐dependency questionnaires, we refer to this first dimension of dependency as passive–submissive dependency. It is important to note that, while passive–submissive dependency stems from traditional views on dependency as a rather stable personality characteristic, we consider the passive and submissive stance of patients as something that can be elicited or reinforced by the specific mental health care context, such as the interaction with their therapist during treatment.

In addition to the traditional link between dependency and passivity, dependent patients also have been described as “clinging,” meaning that they place intense and inappropriate demands on their therapists (e.g., Bornstein, 2005; Clemens, 2010; Hirschfeld et al., 1977). In line with these demands, dependent people can actually be quite active and assertive, depending on the context they are in (Bornstein, 2005). Based on the findings of Morgan and Clark (2010) we call this second dimension of dependency active‐emotional dependency, referring to patients’ emotional neediness in the relationship with the therapist, resulting in an active seeking out for the therapist for emotional support and physical presence. Thus, while dependent patients can be passive in terms of initiative taking in treatment, patients can be quite active in the way that they want to stay close to the therapist and want to continue treatment.

While these two dimensions of dependency–passive–submissive and active‐emotional–have received some theoretical attention, we argue that a third dimension of care dependency may be crucial for obtaining a better understanding of the construct. Specifically, we propose that dependency is intrinsically associated with a lack of perceived attractive alternative options. The notion of the lack of perceived alternatives as a critical aspect of dependency is rooted in interdependence theory (Kelley & Thibaut, 1978; Rusbult, Martz, & Agnew, 1998; Rusbult, Olsen, Davis, & Hannon, 2005), which describes dependency in an interpersonal relationship as the view of one or both partners that they are lacking attractive alternatives to gain the same outcomes. Applying this notion to the domain of mental health care, patients receiving treatment may perceive that in order to fulfil certain desired needs (e.g., a decrease in distress, higher levels of psychological well‐being) they are dependent on their treatment or therapist. Put differently, patients may feel that there are no other or no better alternatives available to fulfil these needs. In the context of mental health care, alternatives may include another therapist, a different type of treatment, support of the partner, family, or friends, or the option to rely on one's own abilities to reduce symptoms.

The lack of perceived alternatives as a third dimension of care dependency helps to explain how patients who receive a psychological treatment become (more) dependent on their treatment via the so called “derogation effect.” Research findings show that when people are in an intimate relationship, they tend to devaluate other possible partners (e.g., Johnson & Rusbult, 1989; for a brief overview, see Lydon & Karremans, 2015). We suggest that a similar derogation effect may occur in a mental health care context. Receiving the help and support of a professional therapist, patients may start to perceive other (nonprofessional) options, such as the support of friends and family, as less attractive or less valuable, whereas the therapist becomes a “guide through life” (Boisvert & Faust, 2002; Linden, 2013). Research findings of Leitner and colleagues are in line with this reasoning. They identified a “dependency/isolation” factor describing a risky development in psychotherapy, referring to patients’ beliefs that the therapist is the most important person in life, and that they do not get the help and support from their network beyond the therapy (Leitner et al., 2013). Others have also theorized about the association between care dependency or mental health care use and patients’ unsatisfactory support systems (Albert, Becker, McCrone, & Thornicroft, 1998; Berk & Parker, 2009; Boisvert & Faust, 2002; Tsai et al., 2012).

## 2 CURRENT STUDY

To summarize, the current study has two goals. The first goal is to develop a theory‐driven questionnaire based on the three hypothesized dimensions of care dependency (i.e., passive–submissive dependency, active‐emotional dependency, and the lack of perceived alternatives). The first part of the paper describes the development, validity, and reliability of this new instrument in a large patient sample receiving psychological treatment. We expect moderate to strong positive intercorrelations for the three potential care dependency dimensions as well as all dimensions to load on one underlying factor. Also, we expect strong positive correlations between the care dependency dimensions and patients’ self‐proclaimed dependency on the treatment (convergent validity) and positive but weak correlations between the three care dependency dimensions and other (trait) dependency measures (discriminant validity). In addition, the test–retest reliabilities of the questionnaire are presented, which are assessed in a second smaller patient sample.

The second goal of the current study is to provide an initial exploration of several correlates of care dependency. The second part of the paper describes the correlations between care dependency and patients’ perceived social support, the quality of the therapeutic alliance, patients’ levels of remoralization, and symptom severity. In line with the view that care dependency is a negative side‐effect of the treatment, we expect all dimensions of care dependency to be related to lower levels of perceived social support, to a lower quality of the therapeutic alliance, to lower levels of remoralization, and to higher levels of psychiatric symptoms. Investigating these associations should increase our understanding of care dependency on a conceptual level and should give some initial clues about whether care dependency is an unwanted and dysfunctional side‐effect of mental health care and psychological treatment, or whether care dependency is actually part of a helpful therapeutic relationship and perhaps even necessary for a psychological treatment to be effective.

## 3 DEVELOPMENT PATIENT SELF‐REPORT QUESTIONNAIRE CARE DEPENDENCY (PART 1)

### 3.1 Method

#### 3.1.1 Participants and procedure

The study took place in collaboration with Pro Persona, a large mental health care institution in the east of The Netherlands. In the current study, adult patients with an outpatient treatment in primary mental health care (PHC) were recruited as well as patients from three different outpatient secondary (specialized) mental health care (SMHC) programs, namely mood disorders, anxiety disorders, and personality disorders. The inclusion criteria for the present study were 1) currently receiving any kind of psychological outpatient treatment (such as cognitive‐behavioral therapy (CBT) or schema‐focused therapy) within the PHC or one of the three SMHC‐programs, and 2) being registered with an e‐mail address in the digital “NETQROM” system, a software program used within Pro Persona to evaluate treatment progress by means of Routine Outcome Monitoring.

Of the 3,552 patients meeting inclusion criterion 1 at the time of the study, 2,949 patients also met inclusion criterion 2. These 2,949 patients were approached by e‐mail including an information letter about the study and a request to participate in the study. If patients agreed to participate they were led to an internet link referring them to an online informed consent form and subsequently to the online version of the questionnaires. Nonresponders received a reminder after 2 weeks. After 2 weeks 853 patients responded, of which 772 gave permission via the informed consent form to participate. This number of 772 patients was 26.2% of the 2,949 patients who were initially contacted by e‐mail. Response rates varied between 17.5% (PHC) to 29.1% (SMHC – Personality disorders). A total of 30 patients were excluded from the sample because they had not reached 18 years of age yet (*n* = 4) or they were still on the waiting list and thus not yet receiving treatment (*n* = 26). The final sample of 742 patients was used in the analyses below.

The 742 patients were on average 41.1 years old (standard deviation (SD) = 11.0; range 18–72). Most of the patients were female (64.6%). With regard to their household, 34.3% reported being single, 28% lived with a partner and one (or more) child(ren), 20.6% lived with a partner without any children, 9.3% lived without a partner but with one (or more) child(ren) living at home, and 7.6% reported another type of household (e.g., living with one or two parents). Of the participants, 51.6% were unmarried; 33% were married, 14% were divorced, and 1.3% were widow(er). With regard to the educational background, 5.9% finished only primary school, 69.7% finished secondary education at a lower or intermediate level, and 24.3% finished higher and/ or scientific education. And 41% of the participants reported to have a paid job.

All patients were receiving a psychological treatment and most patients (88.5% of the final sample) were treated in one of the SMHC‐programs (mood disorders: *n* = 180, 24.3%; anxiety disorders: *n* = 128, 17.3%; personality disorders: *n* = 348, 46.9%, vs. PHC: *n* = 86, 11.6%). More than 95.3% of the patients received an outpatient psychological treatment, and 76.8% of all patients reported to receive an individual treatment, versus 9.6% receiving group therapy, and the remaining 13.5% receiving a combination of individual and group sessions. Moreover, a total of 33.3% of all participants received some form of medication for their symptoms in addition to the psychological treatment. Treatment duration (i.e., the total number of registered sessions a patients has had during the current treatment until the moment the study took place) was quite diverse; 25% of all participants had 15 sessions or less, another 25% of the participants had between 16 and 50 sessions, the next 25% had between 51 and 173 sessions, and the last 25% had more than 173 sessions. A large majority of the participants in the current study, 81.7%, had received at least one psychological treatment previously, of which 53.4% had three or more previous psychological treatments.

Patients completed the digital versions of the questionnaires at home. It took the participants on average 30 minutes to complete all questionnaires. The additional patient‐ and treatment information (e.g., demographics, symptoms severity, and treatment duration) stemmed from questionnaires that were part of the institutions’ Routine Outcome Monitoring and electronic patients’ files.

#### 3.1.2 Test–retest study

Adult patients of an academic outpatient center were asked to participate in the follow‐up study. The center offers primary and secondary mental health care for a general population of outpatients. Offered treatments are mostly based on CBT and a part of their patients are treated within treatment outcome research programs. As the aim of this study was to determine the test–retest reliability of the questionnaire, therapists asked their patients to participate but only if they were currently receiving treatment with an expected between‐session interval of a maximum of two weeks. If patients decided to participate they had to fill out an informed consent form. They were asked to complete the paper versions of the questionnaire after two consecutive treatment sessions.

A total of 32 patients participated, but only 25 patients completed the questionnaires with a between‐session interval of one or two weeks. Of these 25 patients 18 were female and seven were male. Participants were 34.7 years old (SD = 15.1; range 21–68). At the time of the study patients had on average 15.0 treatment sessions (SD = 21.5; range 1–95). Patients reported to be diagnosed with various DSM‐IV main diagnoses: mood disorders (*n* = 8), anxiety disorders (*n* = 7), impulse‐control disorders (*n* = 2), personality disorder (*n* = 1), eating disorder (*n* = 1), others (*n* = 3), or no diagnosis (*n *= 3).

#### 3.1.3 Measures

##### Care dependency

Based on the literature 41 face valid items^1^ were formulated to develop a Dutch self‐report questionnaire supposed to measure the three proposed dimensions of care dependency. As the words “care dependency” could possibly lead to (negative) associations in participants, the questionnaire was called “Therapy experience.” The initial version of the questionnaire consisted of 13 items measuring patients’ passive–submissive dependency (e.g., “I need advice from my therapist when I have to make a decision”); 15 items measuring an active‐emotional dependency (e.g., ”I dread ending the contact with my therapist at the end of the treatment”); and 13 items measuring the lack of perceived alternatives (e.g., "This treatment is the only thing I can hold on to when it comes to tackling my complaints”). Items were rated on a seven‐point Likert scale from (1 = totally disagree, 2 = disagree, 3 = slightly disagree, 4 = neutral, 5 = slightly agree, 6 = agree, 7 = totally agree).

As a first step the 41‐item version of the questionnaire was piloted among 36 adult patients of an academic outpatient center with a variety of disorders. Based on their responses and feedback, five items were removed and 13 items were reformulated because of the following reasons: The item was not formulated clear enough according to the patients; patients reported that two items showed too much overlap with regard to their formulation; the item showed too small variation in their responses (e.g., standard deviation < 1); and/or the item showed negative inter‐item correlations with other items. The remaining 36‐items version was used in the main study as described above (passive–submissive dependency 12 items; active‐emotional dependency 13 items; and lack of perceived alternatives 11 items).

##### Care dependency single‐item

To measure patients’ explicit self‐proclaimed care dependency, the following single‐item was used: “I am dependent on my treatment.” Responses were given on a seven‐point Likert scale (1 = totally disagree, 2 = disagree, 3 = slightly disagree, 4 = neutral, 5 = slightly agree, 6 = agree, 7 = totally agree).

##### Dependent personality disorder symptoms

To measure the degree in which patients suffered from symptoms of a dependent personality disorder, the Dependent Personality subscale of the revised Personality Diagnostic Questionnaire was used (PDQ‐R‐DP; Hyler, Rieder, Williams, Spitzer, Hendler, & Lyons, 1988; Hyler, Rieder, & Williams, 1987). The PDQ‐R‐DP contains a general introductory phrase (“Over the past few years …”) followed by nine statements (e.g., “let I take others important decisions for me”) and a dichotomous yes/ no scale. Usually, the PDQ‐R‐DP is used as a screener for personality disorders by using a cut‐off score, but as we were interested in the number of self‐reported symptoms as validation for our care dependency questionnaire, we used the sum scores of the PDQ‐R‐DP as a continuous measure. Positively formulated items were reversed coded, so that a higher score on the PDQ‐R‐DP indicated more self‐reported symptoms of the dependent personality disorder. Cronbach's alpha in the current sample was rather low (.61).

##### Trait dependency

In order to measure interpersonal dependency as a personality trait, two subscales of the Autonomy‐Connectedness Scale (ACS‐30), developed by Bekker and van Assen (2006), were used. Psychometric properties of the ACS‐30 are acceptable (Bekker & van Assen, 2006). The first subscale, self‐awareness consisted of seven items measuring “the capacity to be aware of one's own opinions, wishes and needs, and the capacity to express these in social situations” (Bekker & van Assen, 2006, p. 52) (e.g., “Usually it is very clear to me what I like most”). Higher scores on this subscale indicated higher self‐awareness (i.e., lower dependency). Cronbach's alpha in the current sample was .81. Sensitivity to others consisted of 17 items and was defined as “a sensitivity to the opinions, wishes, and needs of other people, empathy, and capacity and need for intimacy and separation” (Bekker & van Assen, 2006, p. 52) (e.g., “I often go deeply into other people's feelings”). Higher scores on this subscale indicated a higher sensitivity for others (i.e., higher dependency). Cronbach's alpha in the current sample was .82. All items were rated on a five‐point Likert scale (1 = disagree, 2 = disagree slightly, 3 = disagree slightly and agree slightly, 4 = agree slightly, 5 = agree).

#### 3.1.4 Data analyses

First, data were inspected at item level to determine whether the items were discriminative enough (SD ≥ 1.00), and whether the responses on each item were normally distributed. To determine the reliability of the self‐report questionnaire, Cronbach's alpha's was determined for each of the three theoretical dimensions of care dependency. As we tried to create a questionnaire as reliable but also as short as possible for future use in clinical practice and research, we decided to remove items if this resulted in equal or higher levels of Cronbach's alpha on the particular dimension. To verify whether the remaining items of one dimension fitted into one underlying factor, exploratory factor analyses (EFA, i.e., Principal Axis factoring (PAF)) were performed on each of the three dimensions separately in which the number of factors was set to one. This approach (i.e., conducting separate EFA's per dimension) was unconventional as it is more common to include all items simultaneously within one EFA. However, in the current study the items were formulated for each of the three dimensions separately and independently. Also, the dimensions stemmed from different theoretical approaches (personality psychology, clinical psychology, and social psychology). Therefore, the first logical step was to determine whether each of the three pools of items measured one unidimensional underlying factor. The next step was to examine how the different dimensions were related to each other by investigating the intracorrelations and by performing a second order factor analysis to determine whether one underlying construct “care dependency” could be postulated.

As Kaiser–Meyer–Olkin was sufficient (≥.79) for all dimensions, PAF was suitable to find reliable and distinct factors (Field, 2009; Tabachnick & Fidell, 2007). Scree plots in combination with parallel analysis (PA) were used to decide whether the items of each care dependency dimension were unidimensional, or whether multiple components had to be extracted (O'Connor, 2000). In short, PA is a method in which eigenvalues of components based on observed data are compared to eigenvalues of parallel components derived from random data. A component is retained if the eigenvalue of the *i*th component from the observed data is greater than the 95th percentile eigenvalue of the *i*th component derived from the random data (O'Connor, 2000). As a next step, correlations between the care dependency dimensions and patients’ self‐proclaimed dependency, symptoms of a dependent personality disorder, and trait dependency (i.e., self‐awareness and sensitivity to others) were determined in order to establish convergent and discriminant validity (i.e., moderate [r ≥ .30] to high [r ≥ .50] correlations for convergent validity, in combination with clearly lower to negligible correlations for discriminant analysis, Cohen, 1992; Gregory, 2007). At last, test–retest reliability was determined by examining the Pearson's correlations between the pre‐ and postmeasurements in the second smaller patient sample.

### 3.2 Results

All 36 items of our questionnaire showed sufficient standard deviations except for one item. This item was removed from the analysis. In addition, visual inspection of the histograms showed that not all items were normally distributed: In about half of the items response category “3” (i.e., “slightly disagree”) was chosen less frequently, which resulted in not‐normal distributions. For this reason, PAF was used as the extraction method in the factor analyses, because PAF is the most robust option when assumptions of normality are violated (Fabrigar, Wegener, MacCallum, & Strahan, 1999). In addition, it was decided to only label the two extreme responses at both ends of the seven‐point Likert scale and not the responses in between, to avoid possible “label biases,” aiming for more normal distributed responses on the items. The adapted response scales were used in the test–retest study.

#### 3.2.1 Reliability and factor analyses

##### Passive–submissive dependency

Cronbach's alpha of the initial scale including all 12 items was .73 and could be increased to .74 by deleting one item. Factor analysis (PAF) on these 11 items resulted in a scree plot clearly indicating two distinguishable factors, supported by the results of the PA. Factor 1 explained 28.2% of the variance and included five items measuring the submissive stance of the patient, referring to patients’ need for guidance of the therapist (e.g., “I need advice from my therapist when I have to make a decision”; Cronbach's alpha = .74). The second factor explained 17.9% of the variance and included six items measuring the passive stance of the patient, referring to patients’ lack of confidence to solve problems on their own, and the desire for the therapist to take over the initiative in the treatment (e.g., ”I have difficulty deciding how best to tackle my complaints”; Cronbach's alpha = .71). The correlation between these two factors was .33.

##### Active‐emotional dependency

Cronbach's alpha of the initial scale including all 12 items was almost .86, and increased slightly to .86 after deleting three items. PAF on the remaining nine items resulted in an ambiguous scree plot indicating one or possibly two underlying factors, while PA suggested two different factors. Factor 1 explained 48.6% of the variance and included five items measuring the closeness of the emotional bond with the therapist (e.g., “My therapist really understands me”; Cronbach's alpha = .83). These items were relatively “neutral”, in that they seemed to measure a normal or healthy relationship with the therapist. Factor 2 explained 16.0% of the variance and included four items measuring the patients’ need to be or stay in contact with the therapist (e.g., “The thought of ending the contact with my therapist after the treatment scares me”; Cronbach's alpha = .84). The items of the second factor did have the dependent overtone as intended by the development of this questionnaire. The correlation between the two factors was .58.

##### Lack of perceived alternatives

Cronbach's alpha of the initial scale including all 11 items was .83 and could be increased to .86 by deleting two items. PAF on the remaining nine items resulted in a scree plot and PA clearly indicating one underlying factor, with all items loading ≥ .50 on this particular factor.

#### 3.2.2 Test–retest reliability

Test–retest reliabilities were assessed for the five potential subscales (submissive dependency five items; passive stance six items; emotional bond five items; need for contact four items; lack of perceived alternatives nine items) in the second, smaller patient sample. The test–retest reliabilities (Pearson's correlations) were acceptable for all five subscales of care dependency.

Table 1 offers an overview of the provisional 29 items of the questionnaire. Please note that it presents a translated version of the original Dutch questionnaire. Table 2 offers an overview of the number of items per potential subscale, Cronbach's alpha's, test–retest reliabilities, and other relevant descriptive statistics for all dimensions. Additional item statistics, such as factor loadings and Cronbach's alpha's if item deleted, can be found in the supplemental materials online.

**Table 1:** Overview of the 29‐item version of the care dependency questionnaire and their relevant subscales (Part 1)

#	Item	Subscale
*1*	I come up with my own suggestions and ideas during the sessions with my therapist^a^^,^ b	*Passive stance*
2	I dread ending the contact with my therapist at the end of the treatment	Need for contact
3	In my opinion, this treatment is the only way of ridding myself of my complaints	Lack of perceived alternatives
4	I do not like taking the initiative myself during meetings with my therapist^b^	*Passive stance*
5	My therapist cares about me^b^	*Emotional bond*
6	Also without the help of my therapist, I think I can tackle my problems^a^	Lack of perceived alternatives
7	I present all my decisions to my therapist	Submissive dependency
8	When I am with my therapist, I can be myself^b^	*Emotional bond*
9	Apart from this treatment, I do not see any other options for tackling my problems	Lack of perceived alternatives
10	My therapist ensures that I do not make any wrong choices in my life	Submissive dependency
11	I will miss the contact with my therapist once the treatment has finished	Need for contact
12	I live from treatment session to treatment session	Lack of perceived alternatives
13	During the treatment, I take the initiative myself to tackle my complaints^a^^,^ b	*Passive stance*
14	I have a need for contact with my therapist	Need for contact
15	Without this treatment, my problems will continue to exist	Lack of perceived alternatives
16	When it comes to tackling my complaints or problems, I do not dare to trust my own judgement^b^	*Passive stance*
17	I feel no connection with my therapist^a^^,^ b	*Emotional bond*
18	Only my therapist can help me with my problems	Lack of perceived alternatives
19	I need advice from my therapist when I have to make a decision	Submissive dependency
20	I have a close bond with my therapist^b^	*Emotional bond*
21	Without my therapist I would grind to a halt in the things that I do	Lack of perceived alternatives
22	Actually, my therapist knows better than I do what is good for me	Submissive dependency
23	The thought of ending the contact with my therapist after the treatment scares me	Need for contact
24	Only my therapist can ensure that I keep going	Lack of perceived alternatives
25	It is probably best if my therapist takes the initiative in the meetings^b^	*Passive stance*
26	My therapist really understands me^b^	*Emotional bond*
27	This treatment is the only thing I can hold on to when it comes to tackling my complaints	Lack of perceived alternatives
28	When I make a decision, I consider what my therapist would advise me to do	Submissive dependency
29	I have difficulty deciding how best to tackle my complaints^b^	*Passive stance*

*Note*. Please keep in mind that the original questionnaire that was used on the current studies is in Dutch. This table shows the English adaptation based on a "forward‐and‐backward” translation procedure by professional translation office including a native English speaker. All items were rated on a 1–7 point Likert scale from 1 (completely disagree) to 7 (fully agree).

^a^Item reversed scored. ^b^item not part of final 18‐item version of the questionnaire.

**Table 2:** Number of items; Cronbach's alpha; mean; standard deviation; minimum and maximum; and test–retest correlation for the dimensions of care dependency and the total score (Part 1)

(Sub)scale	# items	α	M^a^	SD^a^	Minimum^a^	Maximum^a^	r_t1,t2_ b
Submissive dependency	5	.74	4.05	1.08	1.00	6.80	.78**
Passive stance^c^	6	.71	3.81	0.95	1.00	6.83	.79**
Emotional bond^c^	5	.83	4.77	1.12	1.00	7.00	.75**
Need for contact	4	.81	4.31	1.39	1.00	7.00	.76**
Lack of perceived alternatives	9	.86	4.19	1.10	1.00	7.00	.80**
Care dependency (total score)^d^	18	.91	4.18	1.01	1.22	6.67	.80**

*Note*. *N* = 742. *r*_t1, t2_ = Test–retest Pearson correlations with a maximum 2‐week interval.

^a^Potential range 1–7. ^b^ *N* = 25. ^c^Passive stance and emotional bond were excluded from the final questionnaire. ^d^The total score is based on the three dimensions submissive dependency, need for contact, and the lack of perceived alternatives.

**p < .001

#### 3.2.3 Correlations

Table 3 presents Pearson's correlations between the five potential subscales of care dependency, the total score of care dependency, as well as the correlations with the other dependency measures. Results showed that the five potential subscales of care dependency –submissive dependency, passive stance, emotional bond, need for contact, and the lack of perceived alternatives–were all, except one, positively correlated with each other, ranging from a weak correlation between the need for contact and patients’ passive stance (*r* = .16) to a strong correlation between submissive dependency and the lack of perceived alternatives (*r* = .66). The only exception was that the emotional bond and patients’ passive stance were negatively correlated to each other (*r* = −.16). All five care dependency potential subscales were also positively associated with patients’ self‐proclaimed dependency on the treatment, with the lack of perceived alternatives showing the strongest correlation (*r* = .60), and passive stance and emotional bond the weakest correlations (*r* = .24). Patients’ passive stance on the other hand was strongest correlated with patients’ self‐reported criteria of a dependent personality disorder, showing a moderate correlation (*r* = .35). The two subscales of the Autonomy‐Connectedness scale, measuring trait dependency, showed that sensitivity for others was only weakly correlated with the need for contact (*r* = .23) and with the lack of perceived alternatives (*r* = .10), while self‐awareness was strongest negatively correlated with patients’ passive stance (*r *= −.43).

**Table 3:** Pearson correlations between the dimensions of care dependency and study variables (Part 1)

	2	3	4	5	6	7	8	9	10
1. Submissive dependency	.24**	.47**	.51**	.66**	.81**	.40**	.20**	.05	−.21**
2. Passive stance^a^	–	− .16**	.16**	.34**	.31**	.24**	.35**	.04	−.43**
3. Emotional bond^a^		–	.52**	.42**	.53**	.24**	.03	.08	.03
4. Need for contact			–	.63***	.80**	.44**	.27**	.23**	−.21**
5. Lack of perceived alternatives				–	.94**	.60**	.25**	.10*	−.22**
6. Care dependency (total score)^b^					–	.58**	.28**	.14**	−.25**
7. Care dependency single‐item						–	.22**	.14**	−.24**
8. Dependent personality disorder symptoms							–	.48**	−.58**
9. Trait dependency – sensitivity for others								–	−.36**
10. Trait dependency – self‐awareness									–

*Note. N *= 742.

^a^Passive stance and emotional bond were excluded from the final questionnaire. ^b^Care dependency (total score) is based on the three subscales submissive dependency, need for contact, and the lack of perceived alternatives.

*p < .01. **p < .001.

#### 3.2.4 Second‐order factor analysis

The second‐order analysis (PAF with promax rotation) on the five potential subscales showed two underlying factors. The first factor explained 53.4% of the variance and included four of the five subscales, i.e., the need for contact, lack of perceived alternatives, submissive dependency, and emotional bond. The second factor explained 23.4% of the variance and only included patients’ passive stance. The two factors were only weakly correlated (*r* = .16).

### 3.3 Preliminary discussion (part 1)

In the first part of the current study, we developed a theory‐driven questionnaire to measure care dependency, based on the three hypothetical dimensions passive–submissive dependency, active‐emotional dependency, and the lack of perceived alternative options. Results showed that the passive–submissive dimension as well as the active‐emotional dimension actually covered two separate underlying factors. With regard to passive–submissive dependency, one factor captured the items measuring patients’ submissiveness in treatment, and the other factor captured patients’ passive stance and the lack of initiative in treatment. The items measuring patients’ passive stance were only weakly to moderately correlated to the other four care dependency dimensions and weakly to patients’ self‐proclaimed dependency (i.e., low convergent validity). Moreover, patients’ passive stance in the second‐order factor analysis was separated as a distinct construct from the general factor underlying the other four dependency subscales. For these reasons, it was decided to exclude patients’ passive stance as part of our care dependency construct and thus to exclude the relevant items from the questionnaire.

With regard to active‐emotional dependency, also two factors emerged. The first one captured the patients’ emotional bond or closeness with the therapist and the other factor one captured the patients’ need to stay in touch with the therapist and fearing the end of the contact after treatment termination. On hindsight we argue that the items from the first factor, the emotional bond, do not measure the dependent aspect of the therapeutic relation as intended while developing this questionnaire, but actually seem to measure a normal or healthy bond with the therapist. This impression was reinforced by the finding that this factor was also only weakly correlated to patients’ self‐proclaimed dependency (i.e., low convergent validity), in contrast to the other subscales of dependency. Therefore, it was decided to exclude this subscale and the relevant items from the questionnaire as well. Both exclusions resulted in a final 18‐item version of the questionnaire measuring care dependency, comprising three unidimensional subscales, namely submissive dependency, need for contact, and the lack of perceived alternatives. In accordance with our expectations, the remaining three care dependency subscales were all moderately to strongly correlated with each other and to patients’ self‐proclaimed dependency (i.e., sufficient convergent validity) and showed only limited overlap with symptoms of a dependent personality disorder and trait dependency (i.e., sufficient discriminant validity).

## 4 PRELIMINARY EXPLORATION OF CORRELATES OF CARE DEPENDENCY (PART 2)

The goal of the second part was to explore the associations between care dependency and several other constructs which appear suited for a better understanding of care dependency and its relevance for clinical practice. The selected constructs in the present study were perceived social support, perceived quality of the therapeutic alliance, patients’ feelings of remoralization, and severity of symptoms. In line with the view that care dependency is a negative side‐effect of mental health care treatments, we expected that higher levels of care dependency were related to lower levels of social support, to a lower quality of the therapeutic alliance, to lower levels of remoralization, and to higher levels of psychiatric symptoms. Results of this second part of the study were based on the same participants (*N* = 742) and study procedure as described in part one of this paper.

### 4.1 Method

#### 4.1.1 Measures

##### Perceived social support

Perceived social support was measured with the Multidimensional Scale for Perceived Social Support (MSPSS; Zimet, Dahlem, Zimet, & Farley, 1988). The MSPSS consists of 12 items measuring perceived support from friends, family, and significant others (e.g., “I get the emotional help and support I need from my family”). Items were rated on a seven‐point Likert scale (1 = very strongly disagree, 2 = strongly disagree, 3 = slightly disagree, 4 = neutral, 5 = mildly agree, 6 = strongly agree, 7 = very strongly agree). The MSPSS has been shown to have good psychometric properties (Zimet et al., 1988; Zimet, Powell, Farley, Werkman, & Berkoff, 1990). Cronbach's alpha in the current sample was .92.

##### Therapeutic alliance

To measure the quality of therapeutic alliance as perceived by the patient, a Dutch adaptation of the patient version of the short Working Alliance Inventory (WAI‐12) was used. The original 36‐item therapist and patient versions of the WAI were developed by Horvath and Greenberg (1989) measuring three components of the working alliance between therapist and client: agreement on the relevance and efficacy of the tasks within in the treatment; agreement on the treatment goals that are endorsed and valued; and the bond between therapist and patient, referring to a positive personal attachment, mutual trust, and acceptance (Horvath & Greenberg, 1989; Dutch adaptation Vertommen & Vervaeke, 1990). In the short version, each dimension was measured by four items and items were scored on a five‐point Likert scale (1 = rarely or never, 2 = sometimes, 3 = often, 4 = very often, 5 = always) (Hatcher & Gillaspy, 2006). The Dutch short adaptation has been shown to have good validity and reliability (Stinckens, Ulburghs, & Claes, 2009). Cronbach's alpha's in the current sample for the tasks‐, goals‐, and bond‐subscales were .89; .87; and .86 respectively.

##### Remoralization

The Remoralization Scale (RS‐12) was used to measure patients’ levels of remoralization (Vissers, Keijsers, van de Veld, de Jong, & Hutschemaekers, 2010). Remoralization refers to feelings of having high self‐esteem, being competent, having a sense of mastery, optimism, inner control, and hope, and can be seen as the inverse of demoralization (Vissers et al., 2010). The RS‐12 items and has been shown to have good psychometric properties (Vissers et al., 2010). Items (e.g., “I am in control of my life”) were responded on a four‐point Likert scale (1 = totally disagree; 2 = disagree a lot; 3 = agree a lot; 4 = totally agree), and higher scores reflected higher levels of remoralization. Cronbach's alpha in de current sample was .94.

##### Symptom severity

To measure the severity of psychiatric symptoms the Dutch adaptation (De Jong, Nugter, Lambert, & Burlingame, 2009) of the Outcome Questionnaire was used (OQ‐45, Lambert et al., 1996). The OQ‐45 consisted of 45 items and covers three domains of dysfunction: symptomatic distress, interpersonal relations, and social role (e.g., “I feel no interest in things,” ”I get along well with others,” "I feel stressed at work/ school”). Items were rated on a five‐point Likert scale (0 = never, 1 = rarely, 2 = sometimes, 3 = often, 4 = almost always) and positively formulated items were reversed scored so that higher scores indicated a higher level of dysfunction. The reliability and validity of the Dutch translation of the OQ‐45 have shown to be adequate to good (de Jong & Nugter, 2004). Cronbach's alpha's in the current sample was .94.

### 4.2 Results

To determine the associations between the study variables, Pearson's correlations between the three dimensions of care dependency, the total score of care dependency, and the other constructs were determined. Table 4 shows the descriptive statistics of all relevant study variables, for the total group as well as separately for the four care programs. Table 5 shows the correlations between the study variables.

**Table 4:** Descriptive statistics of study variables (Part 1 and 2)

		PHC	Mood Disorders	Anxiety Disorders	Personality Disorders	Total
		(*n* = 86)	(*n* = 180)	(*n* = 128)	(*n* = 348)	(*N* = 742)
	Potential Range	M(SD)	M(SD)	M(SD)	M(SD)	M(SD)
Submissive dependency	1–7	4.13(0.89)	4.03(1.19)	4.15(1.08)	4.00(1.07)	4.05(1.08)
Need for contact	1–7	3.89(1.26)	4.31(1.39)	4.44(1.36)	4.37(1.41)	4.31(1.39)
Lack of perceived alternatives	1–7	3.99(0.99)	4.24(1.15)	4.42(1.01)	4.13(1.13)	4.19(1.10)
Care dependency (total score)	1–7	4.00(0.87)	4.20(1.08)	4.35(0.97)	4.15(1.01)	4.18(1.01)
Passive stance	1–7	3.75(0.97)	3.90(0.94)	3.79(0.91)	3.78(0.98)	3.81(0.95)
Emotional bond	1–7	4.93(0.89)	4.61(1.19)	5.03(0.95)	4.71(1.18)	4.77(1.13)
Care dependency single‐item	1–7	4.10(1.57)	4.45(1.85)	4.53(1.50)	4.56(1.69)	4.48(1.69)
Dependent personality disorder symptoms^a^	0–9	4.49(2.15)	5.00(2.05)	5.09(2.07)	5.21(1.97)	5.06(2.04)
Trait dependency – sensitivity to others	1–5	3.62(0.61)	3.71(0.64)	3.87(0.60)	3.92(0.57)	3.83(0.60)
Trait dependency – self‐awareness	1–5	3.11(0.93)	3.03(0.83)	3.02(0.88)	3.01(0.91)	3.03(0.89)
Perceived social support	1–7	4.77(1.29)	4.47(1.34)	4.54(1.26)	4.33(1.39)	4.45(1.35)
Working alliance – tasks	1–5	3.28(0.87)	2.92(0.95)	3.55(0.86)	3.12(1.00)	3.16(0.97)
Working alliance – goals	1–5	3.37(0.85)	2.96(0.96)	3.63(0.80)	3.11(0.97)	3.20(0.95)
Working alliance – bond	1–5	3.31(0.94)	3.06(0.94)	3.41(0.91)	3.15(0.95)	3.19(0.94)
Remoralization	1–4	2.29(0.64)	2.09(0.65)	2.19(0.61)	2.07(0.65)	2.12(0.64)
Symptom severity^b^	0–4	2.49(–)	1.87(0.57)	1.75(0.55)	1.88(0.56)	1.85(0.56)

*Note*. PHC = Primary Health Care.

^a^Sum score. ^b^ *N*_Total_ = 376 because only those patients were selected who completed the OQ‐45 within a 4‐week time period before or after the other questionnaires were administered, resulting in the following sample sizes per care program with regard to the symptom severity measure: *n*_PHC_ = 1; *n*_Mood Disorders _= 113; *n*_Anxiety Disorders_ = 84; *n*_Personality Disorders_ = 178.

**Table 5:** Pearson's correlations between study variables (Part 2)

	1	2	3	4	5	6
Submissive dependency	.05	.32***	.30***	.25***	−.07*	.11*
Need for contact	−.09*	.27***	.26***	.30***	−.19***	.16***
Lack of perceived alternatives	−.12**	.28***	.25***	.21***	−.25***	.24***
Care dependency (total score)	−.08*	.33***	.31***	.28***	−.22***	.21***
Passive stance^a^	−.19***	−.27***	−.28***	−.22***	−.45***	.37***
Emotional bond^a^	.15***	.63***	.62***	.72***	.18***	−.20***
1. Perceived social support	–	.26***	.24***	.24***	.37***	−.39***
2. Therapeutic alliance – tasks		–	.87***	.65***	.37***	−.33***
3. Therapeutic alliance – goals			–	.71***	.32***	−.30***
4. Therapeutic alliance – bond				–	.27***	−.28***
5. Remoralization					–	−.69***
6. Symptom severity^b^						–

*Note*. *N* = 742.

^a^Passive stance and emotional bond were excluded from the questionnaire. ^b^N = 376 because only those patients were selected who completed the OQ‐45 within a 4‐week time period before or after the other questionnaires were administered.

*p < .05. **p < .01. ***p < .001.

#### 4.2.1 Perceived social support

The total score of care dependency was only very weakly related to lower levels of perceived social support (*r* = −.08). At subscale level, a larger need for contact with the therapist as well as a greater lack of perceived alternatives were weakly correlated with lower levels of perceived social support (respectively *r* = −.09 and *r* = −.12), while submissive dependency was unrelated to patients’ social support.

#### 4.2.2 Therapeutic alliance

Moderate positive correlations were found between the three subscales of care dependency and the perceived quality of the therapeutic alliance, indicating that higher levels of patients’ care dependency were related to a better therapeutic alliance. We also explored the correlations between the excluded subscales and therapeutic alliance. Noteworthy is that these two subscales showed different patterns as compared to the remaining care dependency subscales. Emotional bond showed a much stronger positive correlation with the working alliance, especially with the subscale measuring the closeness of the bond between patient and therapist. In contrast, higher levels of patients’ passive stance were related to lower quality of the therapeutic alliance.

#### 4.2.3 Remoralization

Higher levels of dependency were related to somewhat lower levels of remoralization (*r* = −.22), indicating that patients’ who were more *de*moralised also reported higher levels of dependency.

#### 4.2.4 Symptoms severity

Higher levels of dependency were related to somewhat higher levels of symptoms severity (*r* = .21), indicating that patients’ who were more dependent on the treatment also report more psychiatric symptoms and vice versa.

### 4.3 Preliminary discussison (Part 2)

In the second part of the paper, the correlations were explored between the three subscales of care dependency and perceived social support, the quality of the therapeutic alliance, patient's levels of remoralization, and symptom severity. As expected, results showed that in general higher levels of patients’ care dependency were related to higher symptom levels, as well as to lower levels of remoralization (i.e., more demoralization) and to somewhat lower levels of perceived social support. However, against our expectations, higher levels of care dependency were related to a higher instead of a lower quality of the therapeutic alliance as perceived by the patient.

### 4.4 General discussion

The current study had two goals. The first goal was to develop a questionnaire which would be helpful to investigate care dependency according to three hypothesized dimensions. The second goal of the current study was to provide a preliminary exploration of several correlates of care dependency, selected in order to explore whether care dependency should be conceived as an unwanted and dysfunctional side‐effect of mental health care and psychological treatments, or whether care dependency may actually be part of a helpful therapeutic relationship and perhaps even necessary for an effective psychological treatment.

The questionnaire appears to be a reliable measure of three unidimensional subscales of patients’ care dependency, namely submissive dependency (i.e., submissive stance toward the therapist, need for therapists’ guidance and advice), need for contact (i.e., need for contact with the therapist, and dreading to end the contact with the therapist at the end of the treatment), and the lack of perceived alternatives (i.e., perception that only with the help of this particular treatment or therapist their problems can be solved). Based on statistical and conceptual reasons, we decided to omit the patients’ passive stance and the need for contact from our questionnaire. Both factors hinder a clear operationalization of the concept care dependency. Furthermore, all three remaining subscales could be empirically distinguished from trait dependency and self‐reported symptoms of a dependent personality disorder.

As mentioned earlier, patient dependency in clinical practice is often seen as a personality trait of the patient, which has traditionally been linked to patients being passive, weak, and showing a helpless attitude toward other people in their lives, including their therapist. Our first main finding, however, is that patients’ dependency on their treatment or therapist was only weakly to moderately related to patients’ dependent trait characteristics, strongly suggesting that trait dependency and care dependency are different constructs. While people relying heavily on others in general may also be more prone to develop a dependent stance in treatment, our results suggest that the specific mental health care context plays an additional role in whether or not people become dependent on their treatment. For example, during the psychotherapeutic process decisions may often be left to the therapist, which may reduce perceived self‐efficacy and increase perceived dependency in the patient. Additionally, during the period of therapy, patients may mentally consult their therapist to make decision in everyday life (i.e. “what would my therapist think?”) (c.f. Berk & Parker, 2009). Although such processes are speculative, our findings suggest that care dependency is a real phenomenon that can be distinguished from trait dependency, and it is therefore important for future research to examine the determinants and consequences of care dependency.

The first part of the paper also suggests that the "lack of perceived alternatives” is a useful concept in understanding care dependency and is perhaps even a central aspect of it. The lack of perceived alternatives showed the strongest correlations with the other two subscales of care dependency and was more strongly related to patients’ self‐proclaimed treatment dependency as compared to the other two subscales of care dependency. This particular finding provides a significant contribution to the literature, offering additional ways of understanding why patients become more dependent on their treatment. Similar to dependency in intimate (romantic) relationships, patients in the mental health care context may more or less automatically start to devaluate other alternative sources of help and support when they are in treatment (i.e., derogation effect). Indeed, consistent with this reasoning, we found that a greater lack of perceived alternatives was related to lower levels of perceived social support. However, as this association was only weak it is possible that support from friend and family is not considered as an alternative for a professional treatment (Tsai et al., 2012). Future studies should reveal whether it is indeed the case that patients start to devaluate other alternative options during treatment, and whether this leads to higher levels of dependency as reflected in the other dimensions.

Our findings indicate that care dependency may cover both negative as well as positive features. On one hand, in line with the prevailing negative perspective on dependency, we found that the three subscales of care dependency, i.e., submissive dependency, need for contact, and the lack of perceived alternatives, were related to higher levels of symptoms and lower levels of remoralization (i.e., more demoralization). One interesting possibility is thus that higher levels of care dependency increases patients’ symptom levels and demoralization, which would suggest a negative effect of care dependency.

On the other hand, our findings also hint to potential positive outcomes of care dependency. First, due to the cross‐sectional nature of the study, the association between care dependency and symptoms and demoralization may also suggest that higher care dependency results from higher symptom levels and demoralization. When patients experience more severe psychiatric symptoms, they may become more demoralized, showing lower levels of self‐efficacy, less inner control, and so on. Consequently, as a possible functional response, patients may feel more in need for help and therefore report higher levels of care dependency. Second, and somewhat related, the three subscales of care dependency were positively related to patients’ satisfaction with the alliance with their therapist. As it has repeatedly been demonstrated that patients’ perception of the quality of the therapeutic alliance predicts better treatment outcomes (e.g., Horvath, Del Re, Flückiger, & Symonds, 2011; Lambert & Barley, 2001), it seems plausible that higher levels of dependency as associated with a better therapeutic alliance could also lead to more effective psychological treatments. A possible explanation for this may be that dependent patients are more open for suggestions and ideas of the therapist, resulting in more influential power of the therapist (c.f., Hoyt, 1996; Perrin, Heesacker, Pendley, & Smith, 2010). Patients’ care dependency can be a functional help‐seeking response to psychological suffering, provoked by a good therapeutic alliance between the therapist and patient, and leading to better treatment outcomes due to the increased influence of the therapist on the patient. In sum, however, the question whether care dependency is a functional or dysfunctional aspect of psychological treatments cannot be answered unequivocally, highlighting the need for more systematic research on this topic.

In several ways, our findings suggest that patients’ passive stance in treatment is actually not part of care dependency. Patients’ passive stance was less strongly associated with the other care dependency subscales and also not as strongly related with patients’ self‐proclaimed dependency on the treatment as compared to the other care dependency subscales. Also, the second‐order factor analysis showed that the items belonging to patients’ passive stance measured something different than the items of the other remaining care dependency subscales, and both factors were only weakly correlated to each other. At the same time, passive stance showed the strongest correlations with trait dependency and symptoms of a dependent personality disorder. Moreover, higher levels of patients’ passive stance were stronger related to higher levels of symptoms and demoralization, and, in contrast to the remaining subscales of care dependency, to lower levels of patients’ satisfaction with the therapeutic alliance. Taken together, these findings suggest that patients’ passive stance captures the dependent characteristics of the trait approach to dependency as how it is traditionally viewed in clinical practice (e.g., Bornstein, 2005). However, it does not belong to the concept of patients’ dependency on the psychological treatment. In addition, it seems that especially this passive stance of patients might be dysfunctional in mental health care treatments, and it is therefore important to distinguish patients’ passive stance from patients’ levels of care dependency.

We should mention some limitations of the study. First, the current study used exploratory methods. Before applying this dependency measure in (individual) clinical practice, future research is needed to confirm our findings regarding our dependency subscales (e.g., using confirmatory factor analysis) and regarding the associations with other existing dependency measures and other clinical relevant variables (e.g., combining the dependency subscales in multivariate analyses to test the effect of care dependency on treatment outcomes). Also, the rather low internal consistency of the PDQ‐R‐DP in the current study has to be taken into account, for example by using clinical interviews to determine patients’ symptoms of a dependent personality disorder. Second, the current study is cross sectional, and future studies should provide more insight into the causal relations between the three care dependency subscales, perceived therapeutic alliance, and treatment outcomes. Longitudinal or predictive studies will permit stronger conclusions about whether patients’ passive stance may be a dysfunctional aspect of patients’ trait dependency, while care dependency promotes the effectiveness of psychological treatments. Third, although the naturalistic sample of 742 patients is large and covers the most common disorders in the mental health care in The Netherlands, the sample is also very heterogeneous. It includes patients with various disorders and severity levels, treated in different treatment settings with various forms and lengths of psychological treatments. Also, patients were in different phases of their treatments when they completed the questionnaires. The way care dependency is related to these different patient‐ and treatment characteristics (e.g., the association between dependency and treatment duration), and the way dependency might develop across the course of treatment therefore cannot be addressed with the current data, and remain crucial and important issues for future research.

The present findings open various avenues for interesting future questions to explore. For example, do certain therapeutic approaches or treatment characteristics influence patients’ levels of dependency? And does patients’ dependency in turn predict the duration of their treatment, above and beyond the severity of symptoms? Is there an optimal level or optimal (nonlinear) pattern of dependency during treatment that results in more symptom reduction than other patterns? With the use of our newly developed questionnaire it should be possible to empirically answer such questions. Our hope is that our initial exploration stimulates future research of this important but understudied topic.

## Supporting information

Supplementary Table 1 Descriptive Item Statistics for Passive‐Submissive Dependency Dimension
Supplementary Table 2 Descriptive Item Statistics for Active‐Emotional Dependency Dimension
Supplementary Table 3 Descriptive Item Statistics for Lack of Perceived Alternatives

## NOTE

1 The initial version of the questionnaire included 13 extra items formulated to measure the degree of goal directedness in treatment, resulting in an initial 54‐item version of the questionnaire. However, during the process of developing the questionnaire we believed that these particular items did not suit the goal of this questionnaire (i.e., measuring care dependency) because we hypothesized that the lack of goal directedness should be seen as a potential predictor of care dependency and not as part of the concept itself. As the first analyses also showed negative correlations between this dimension and the other dimensions of care dependency, we chose to exclude these items from our questionnaire in the subsequent steps. For reasons of clarity, we chose not to include these items in the current paper.
