# Supplementary material for: Patients’ care dependency in mental health care: Development of a self‐report questionnaire and preliminary correlates
Source: J Clin Psychol. 2018 Jan 10;74(7):1189–206. doi: 10.1002/jclp.22574 (PMC6619340; doi:10.1002/jclp.22574)
Supplement: Supplementary file 1 — Supplementary Table 1 Descriptive Item Statistics for Passive‐Submissive Dependency Dimension Supplementary Table 2 Descriptive Item Statistics for Active‐Emotional Dependency Dimension Supplementary Table 3 Descriptive Item Statistics for Lack of Perceived Alternatives [file JCLP-74-1189-s001.docx]

Supplementary Table 1

Descriptive Item Statistics for Passive-Submissive Dependency Dimension

| # | Item |  | | Factor Loadings^a^ | |  | |
| --- | --- | --- | --- | --- | --- | --- | --- |
|  |  | *M* | *SD* | Submissive Stance | Passive Stance | *r*_it_^b,c^ | Cronbach’s α if Item Deleted^b^ |
| 19 | I need advice from my therapist when I have to make a decision | 4.72 | 1.48 | **.67** | -.01 | .53 | .68 |
| 28 | When I make a decision, I consider what my therapist would advise me to do | 4.46 | 1.57 | **.65** | -.16 | .49 | .69 |
| 10 | My therapist ensures that I don’t make any wrong choices in my life | 3.37 | 1.60 | **.63** | -.05 | .55 | .67 |
| 22 | Actually, my therapist knows better than I do what is good for me | 3.90 | 1.55 | **.62** | .16 | .52 | .68 |
| 7 | I present all my decisions to my therapist | 3.78 | 1.57 | **.48** | -.05 | .40 | .73 |
| 25 | It’s probably best if my therapist takes the initiative in the meetings^d^ | 3.64 | 1.46 | .06 | **.65** | .55 | .63 |
| 1 | I come up with my own suggestions and ideas during the sessions with my therapist^d,e^ | 3.02 | 1.43 | -.16 | **.60** | .43 | .67 |
| 13 | During the treatment I take the initiative myself to tackle my complaints^d,e^ | 3.23 | 1.34 | -.13 | **.56** | .40 | .68 |
| 4 | I don’t like taking the initiative myself during meetings with my therapist^d^ | 3.60 | 1.69 | -.07 | **.55** | .44 | .67 |
| 16 | When it comes to tackling my complaints or problems, I don’t dare to trust my own judgement^d^ | 4.43 | 1.63 | .28 | **.42** | .42 | .67 |
| 29 | I have difficulty deciding how best to tackle my complaints^d^ | 5.01 | 1.43 | .16 | **.42** | .41 | .68 |

*Note.* N = 742.

^a^Factor loadings based on Principal Axis Factoring with Promax Rotation. Factor loadings over .32 appear in boldface. ^b^Statistics based on separate reliability analysis per factor. ^c^*r*_it_ = part-whole corrected item-total correlation. ^d^item not part of final 18-item version of the questionnaire. ^e^Item reversed scored.

Supplementary Table 2

Descriptive Item Statistics for Active-Emotional Dependency Dimension

| # | Item |  | | Factor Loadings^a^ | |  |  |
| --- | --- | --- | --- | --- | --- | --- | --- |
|  |  | *M* | *SD* | Emotional Bond | Need for contact | *r*_it_^b,c^ | Cronbach’s α if Item Deleted^b^ |
| 5 | My therapist cares about me^d^ | 4.51 | 1.43 | **.76** | -.08 | .64 | .80 |
| 26 | My therapist really understands me^d^ | 5.01 | 1.44 | **.75** | .05 | .70 | .78 |
| 8 | When I’m with my therapist, I can be myself^d^ | 5.28 | 1.40 | **.72** | -.14 | .57 | .82 |
| 17 | I feel no connection with my therapist^d,e^ | 5.27 | 1.54 | **.69** | .04 | .63 | .80 |
| 20 | I have a close bond with my therapist^d^ | 3.76 | 1.43 | **.60** | .19 | .63 | .80 |
| 23 | The thought of ending the contact with my therapist after the treatment scares me | 3.90 | 1.83 | -.15 | **.93** | .73 | .76 |
| 2 | I dread ending the contact with my therapist at the end of the treatment | 4.23 | 1.82 | -.11 | **.79** | .65 | .80 |
| 11 | I will miss the contact with my therapist once the treatment has finished | 4.57 | 1.64 | .25 | **.68** | .73 | .76 |
| 14 | I have a need for contact with my therapist | 4.56 | 1.47 | .16 | **.52** | .56 | .83 |

*Note.* N = 742.

^a^Factor loadings based on Principal Axis Factoring with Promax Rotation. Factor loadings over .32 appear in boldface. ^b^Statistics based on separate reliability analysis per factor. ^c^*r*_it_ = part-whole corrected item-total correlation. ^d^item not part of final 18-item version of the questionnaire. ^e^Item reversed scored.

Supplementary Table 3

Descriptive Item Statistics for Lack of Perceived Alternatives

| # | Item |  |  | Factor Loadings^a^ |  | |
| --- | --- | --- | --- | --- | --- | --- |
|  |  | *M* | *SD* | Lack of Perceived Alternatives | *r*_it_^b^ | Cronbach’s α if Item Deleted^b^ |
| 27 | This treatment is the only thing I can hold on to when it comes to tackling my complaints | 4.08 | 1.70 | .77 | .70 | .83 |
| 21 | Without my therapist I would grind to a halt in the things that I do | 4.26 | 1.59 | .69 | .62 | .84 |
| 24 | Only my therapist can ensure that I keep going | 3.06 | 1.52 | .68 | .62 | .84 |
| 18 | Only my therapist can help me with my problems | 3.39 | 1.57 | .68 | .63 | .84 |
| 15 | Without this treatment my problems will continue to exist | 5.16 | 1.56 | .67 | .62 | .84 |
| 12 | I live from treatment session to treatment session | 3.43 | 1.70 | .59 | .53 | .85 |
| 9 | Apart from this treatment, I don’t see any other options for tackling my problems | 4.09 | 1.74 | .58 | .54 | .85 |
| 6 | Also without the help of my therapist, I think I can tackle my problems^c^ | 5.56 | 1.44 | .54 | .50 | .85 |
| 3 | In my opinion this treatment is the only way of ridding myself of my complaints | 4.66 | 1.73 | .50 | .46 | .85 |

*Note.* N = 742.

^a^Factor loadings based on Principal Axis Factoring. Factor loadings over .32 appear in boldface. ^b^*r*_it_ = part-whole corrected item-total correlation. ^c^Item reversed scored.
